# 2La chromosomal inversion enhances thermal tolerance of *Anopheles gambiae *larvae

**DOI:** 10.1186/1475-2875-8-147

**Published:** 2009-07-02

**Authors:** Kyle AC Rocca, Emilie M Gray, Carlo Costantini, Nora J Besansky

**Affiliations:** 1 Eck Institute for Global Health, Department of Biological Sciences, University of Notre Dame, Notre Dame, Indiana, USA; 2 Institut de Recherche pour le Développement (IRD), UR016, and Laboratoire de Recherche sur le Paludisme, Organisation de Coordination pour la lutte contre les Endémies en Afrique Centrale (OCEAC), Yaoundé, Cameroon

## Abstract

**Background:**

The mosquito *Anopheles gambiae *is broadly distributed throughout sub-Saharan Africa and this contributes to making it the most efficient vector of malaria on the continent. The pervasiveness of this species is hypothesized to originate in local adaptations facilitated by inversion polymorphisms. One inversion, named 2La, is strongly associated with aridity clines in West and Central Africa: while 2La is fixed in arid savannas, the 2L+^a ^arrangement is predominantly found in the rainforest. Ability to survive high temperature exposure is an essential component of aridity tolerance, particularly in immature stages that are restricted to shallow puddles. Toward deciphering the role of the 2La inversion in local adaptation, the present investigation focused on variation in larval and pupal thermo-tolerance in two populations dissimilar solely in 2La arrangement.

**Methods:**

A laboratory colony of *A. gambiae *that is polymorphic for 2La but standard for all other known inversions was used to create 2 homokaryotypic populations (2L+^a ^and 2La). The survival of 4^th ^instar larvae and pupae from both populations was then tested following exposure to thermal stress with and without prior heat hardening.

**Results:**

Larvae responded identically to a 40°C heat stress, with about 50% of larvae dying after 1.5–2 h and few larvae surviving a 3 h stress. When heat hardened prior to the thermal stress, thermo-tolerance of both larval populations increased, with 2La 24 h survival significantly exceeding that of 2L+^a^. Pupae were generally more thermo-tolerant than larvae, although 2La pupae were less so than 2L+^a^. Heat hardening had no positive effect on pupal thermo-tolerance.

**Conclusion:**

The increased thermo-tolerance observed in 2La larvae following heat hardening suggests higher responsiveness (i.e., thermal sensitivity) of the inverted karyotype. By responding more drastically to the heat shock, 2La larvae are better equipped to resist the potentially lethal temperatures that occur in arid habitats. The lower survival of 2La pupae compared with 2L+^a ^may reflect the cost of this sensitivity, whereby the thermal resistance mechanisms prevent successful completion of metamorphosis. The costs and benefits of thermal resistance are discussed in light of the climates characterizing either end of the 2La frequency cline.

## Background

The pervasiveness of malaria throughout sub-Saharan Africa is linked to that of its vector *Anopheles gambiae*, who has successfully invaded and adapted to most ecosystems found on the continent [1]. The impressive geographic and seasonal distribution of this species is hypothesized to originate in local adaptations facilitated by inversion polymorphisms [2]. Indeed, extensive cytogenetic studies have found that *A. gambiae *is not a genetically homogeneous species, but is composed of a mosaic of populations distinguished by different combinations of chromosomal inversions [3]. It is thought that these inversions provide the species as a whole with phenotypic flexibility allowing it to thrive in diverse habitats.

Past research has supported a role for chromosomal inversions in facilitating local environmental adaptation [4]. Inversion clines have repeatedly been observed in *Drosophila *populations [5] and reestablish themselves rapidly following species introduction events [6]. Also, inversions are associated with variation in stress resistance and other phenotypes [4], lending further support to their role in local adaptation. *Anopheles gambiae *also presents clines in inversion frequencies, as has been repeatedly observed along transects ranging from equatorial forests in southern Nigeria and Cameroon to arid savannas in the north [3,7]. It is hypothesized that these inversions are also associated with specific phenotypes that are under differential selection, maintaining the inversion clines and ultimately permitting range expansion of the vector mosquito.

One such inversion (2La) shows a particularly strong association with climate. The 2La arrangement is absent in southern Nigeria and southern Cameroon and increases progressively to reach fixation in the north of these countries [3,7]. Abiotic differences between habitats typical of 2La and 2L+^a ^include mean annual precipitation and diurnal temperature variation. Arid habitats of northern Cameroon for example receive less rain and are subjected to higher maximal and lower minimal temperatures than southern coastal Cameroon. While Douala, in the south, receives over 3,800 mm annual rainfall, Maroua in the north receives only 850 mm (averages over past 103 years) [8]. Average lows/highs are 23/28°C (record 17/37°C) in the south versus 22/35°C (record 11/45°C) in the north. Rainfall and thermal range are both linked to cloud cover: the clouds common in the southern coastal regions bring moisture and create a greenhouse effect dampening diurnal temperature changes. The north experiences less cloud cover, hence is subjected to more intense daily solar radiation, while at night more heat is lost to the clear sky. The climatic conditions of the north result in a set of abiotic stresses – low humidity and temperature extremes – that are likely to drive selection processes in *A. gambiae*. Given the strong 2La frequency cline, it seems likely that this inversion is under selection based on advantages it confers in the arid north [1,3,9,10].

Immature stages are particularly exposed to temperature fluctuations as they live in shallow puddles in open habitats and cannot easily evade unfavorable conditions. Larval habitats have been found to range in temperature from 25 to nearly 42°C [11-13]. The larva's growth rate is proportional to ambient temperature within the bounds of viable temperatures (16–32°C) [14], but significant mortality occurs at higher temperatures, presumably as a result of protein unfolding and the breakdown of physiological processes. Stress response mechanisms exist to protect organisms undergoing acute stress exposure, and act for example by up-regulating heat shock protein (*hsp*) gene expression and altering metabolism. These stress response mechanisms and their temperature sensitivity vary among species and even populations adapted to different environments in order to balance the benefits of the response mechanism and its costs [15,16].

This study aimed to determine whether the 2La chromosomal inversion is associated with enhanced thermo-tolerance in larvae and pupae of the malaria vector *A. gambiae*. The strong association of 2La with hot and thermally variable environments led to the prediction that carriers of 2La will have enhanced thermo-tolerance when compared with their standard 2L+^a ^counterparts. Furthermore, the thermal sensitivity of both chromosomal arrangements was compared following heat hardening, which represents a short exposure to a sub-lethal temperature known to trigger a stress response mechanism.

The ultimate goal is to link physiological differences found among alternative karyotypes with the genetic variation between inverted and standard sequences, currently under investigation in the same laboratory [17]. The present study advances understanding of the vectors' stress tolerance and potential to adapt to new environmental conditions, and when coupled with ongoing molecular approaches, can help elucidate genotype/phenotype correlations relevant to the maintenance of inversion polymorphism [4].

## Methods

### Colonies

Two homokaryotypic strains of *A. gambiae *were created from a colony that is fixed and standard for all inversions on 2R but polymorphic for 2La. The parental colony named SUCAM originated early in 2005 from the cross between CAM (2R+/2L+^a^) and SUA (2R+/2La), both representing the M molecular form of *A. gambiae *derived from regions near Yaoundé, Cameroon and Suakoko, Liberia, respectively. After approximately 36 generations of intermating (assuming 1 generation per month), homokaryotypic sub-strains, named SUCAM 2La and SUCAM 2L+^a ^were created in April 2008 by identifying the 2L karyotype of live adults using DNA from one leg. On the morning of adult emergence, mosquitoes were cold anesthetized, amputated of one rear leg and isolated in a numbered vial. Each leg was ground in lysis buffer [18] and PCR was performed using 2La and 2L+^a ^specific primers [19]. By early afternoon, the 2L karyotype of each mosquito was known, allowing placement of each mosquito in the appropriate cage. Selection was terminated when each population cage contained at least 70 individuals. All mosquito populations were maintained at 27°C and 80% RH and a 12:12 h light-dark cycle. Larvae were reared in shallow plastic trays at a density of approx. 100/L of deionized water, and fed a mixture of 2:1 (by mass) fish food (Tetramin^®^, Germany) and yeast. Experiments were initiated after three generations.

### Thermo-tolerance

Thermo-tolerance of 4^th ^instar larvae and pupae was examined by subjecting individuals to a thermal stress (TS) for various durations. Twenty-five larvae or pupae of each karyotype were individually placed in 13 × 100 mm glass culture tubes with approx. 2 ml water and immersed in a waterbath set at 40°C for t = 30, 60, 90, 120, 150 or 180 min. A control group was treated identically but not subjected to the TS (t = 0 min). Following TS, tubes were returned to the insectary and placed in 27°C water to cool them promptly. Larval survival was assessed 24 h after the TS (immediate mortality response), as was their ability to pupate (delayed mortality response), in order to detect possible effects of the heat stress on survival to adulthood. Pupal survival following TS was assessed by the ability to emerge (pupal mortality response). Each time point for each experiment was repeated six times, bringing the total number of individuals tested to 150 per time point.

### Heat hardening

The effect of heat hardening (HH) on subsequent thermo-tolerance of larvae was examined. HH was performed by placing larvae or pupae in a 38°C water-bath for 60 min, 2 h prior to TS. From the previous experiment, the duration of TS leading to about 50% survival of larvae (120 min) and pupae (150 min) was identified and used to compare survival of heat hardened (HH+TS) and non-heat hardened (TS only) individuals. A control set of individuals (HH only) was only heat hardened to verify that the HH treatment did not in itself impact survival. Survival of all individuals was assessed as above.

### Statistical analyses

The effect of TS duration on survival of both populations was assessed by logistic regression allowing for overdispersion in the data using R v.2.9.0 [20]. The regression line equations were used to derive median and 99% lethal durations, with confidence intervals estimated from the *dose.p *function of the MASS library in R. Statistical inference about main effects of population, TS duration, and their interaction, followed analysis of deviance procedures: after fitting the full model, statistical significance was assessed by the change in deviance resulting from the removal of interaction and main effects, in turn, from the model. To assess the effect of prior HH on the TS response, the odds ratio of the probability of survival between HH+TS and TS, and its standard error, was calculated by logistic regression. The probability of survival was defined based on the mean number of larvae or pupae dying out of the total number exposed.

## Results

### Thermo-tolerance

**Figure 1:**
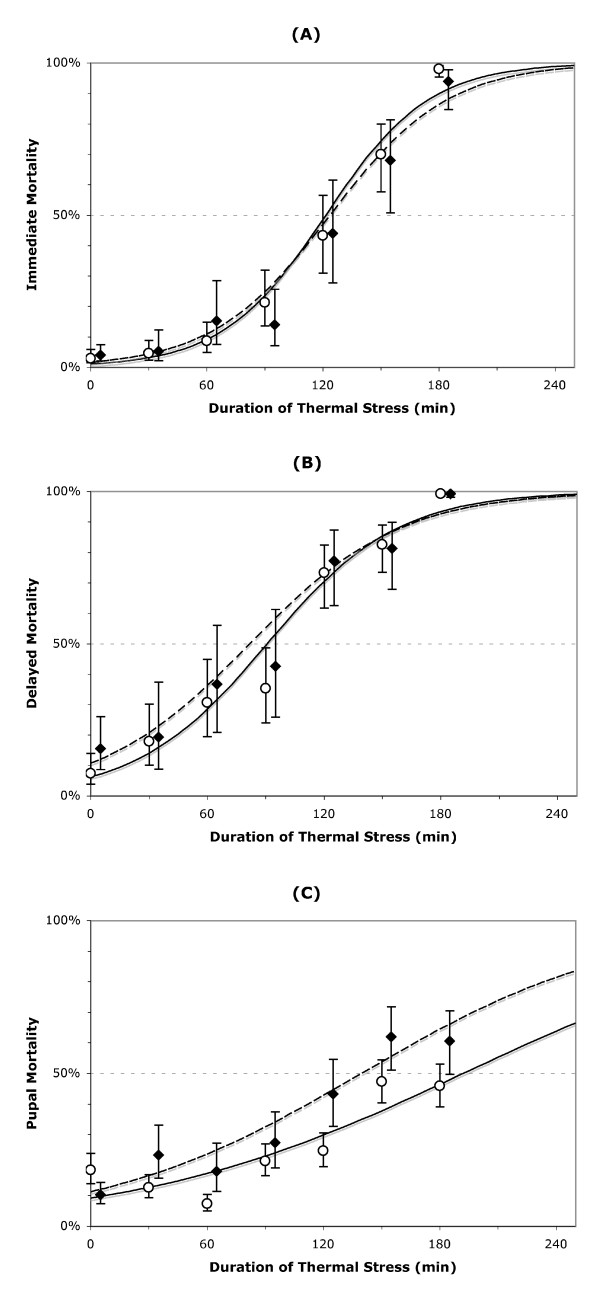
**Survival of *Anopheles gambiae *larvae**. (A: mortality 24 hours post-treatment; B: delayed mortality, i.e. inability to pupate) and pupae (C) following a 40°C thermal stress of variable duration. Open circles and solid lines refer to 2L+^a^/+^a^, and closed diamonds and dashed lines refer to 2La/a karyotypes. Bars represent standard error of the mean of 6 repeats for each time interval. Points referring to 2La/a karyotypes are slightly shifted to the right of the appropriate value on the abscissa for visualization purposes.

Overall, mortality of larvae exposed to a 40°C stress was related to the duration of the exposure (Figure 1A and 1B). Median mortality was estimated at ≈1.5–2 h, rising to 99% mortality after ≈4 h of exposure (Table 1). Immediate and delayed mortality of larvae was not affected by the population karyotype, i.e., survival curves of both populations were nearly identical (Table 2; Figure 1A and 1B). Pupae were generally more resistant than larvae to TS, with median mortality estimated at ≈2.5–3 h exposure (Table 1; Figure 1C). However, 2La pupae were overall significantly less tolerant of the stress by about 0.5 h than 2L+^a ^(Tables 1 and 2).

**Table 1:** Median and 99% lethal durations [95% confidence interval] in minutes after exposure of larvae or pupae to a 40°C thermal stress.

Endpoint	Larvae		Pupae	
		
	Immediate	Delayed	2L+^a^/+^a^	2La/a
LD_50_	123	86	183	143
	[116, 129]	[80, 93]	[161, 205]	[125, 161]
LD_99_	254	253	526	486
	[233, 275]	[229, 277]	[437, 614]	[404, 568]

Figures are a pooled estimate for larvae given that no difference in response was found between karyotypes (see Figure 1 and Table 2).

**Table 2:** Analysis of deviance (ANODEV) for effects of population, thermal stress duration and their interaction on proportion of *Anopheles gambiae *larvae surviving (immediate mortality), pupating (delayed mortality), and pupae emerging (pupal mortality).

	Larvae surviving				Larvae pupating				Pupae emerging			
			
Source	Deviance	d.f.	F	*P*	Deviance	d.f.	F	*P*	Deviance	d.f.	F	*P*
Duration	1144.3	1	171.46	<0.0001	988.5	1	327.18	<0.0001	234.1	1	98.66	<0.0001
Population	0.1	1	0.04	0.841	5.3	1	1.76	0.188	27.5	1	11.59	0.001
Dur × Pop	2.5	1	0.74	0.392	3.1	1	1.03	0.313	2.3	1	0.98	0.325
Residual	288.1	84			266.0	84			188.8	78		

Total	1435.0	87			1260.8	87			449.6	81		

### Heat hardening

**Figure 2:**
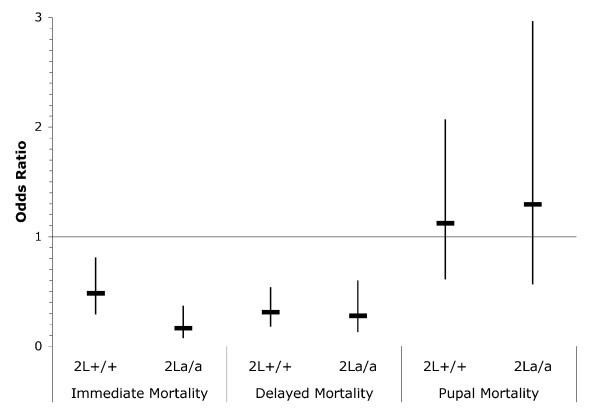
**Effect of prior heat hardening (1 h at 38°C) on the thermo-tolerance of *Anopheles gambiae *larvae (immediate and delayed mortality) and pupae (pupal mortality) following thermal stress treatment**. Mean and 95% confidence interval of the odds ratio for each karyotype.

HH alone resulted in immediate, delayed, and pupal mortality averaging 5.0%, 8.5%, and 13.5%, respectively. HH significantly reduced both immediate and delayed larval mortality following thermal stress, as demonstrated by the mean odds ratio being lower than unity and its 95% confidence interval not overlapping unity (Figure 2). This effect was significantly larger for the 2La population only in the case of immediate mortality (*t *= 5.189, *P *< 0.0001). HH did not improve the survival of pupae to TS (no significant treatment effect) regardless of karyotype (Figure 2).

## Discussion

The effect of thermal stress and heat hardening on survival was studied in *A. gambiae *larvae and pupae differing only in the 2La inversion arrangement. *Anopheles gambiae *are restricted to shallow aquatic habitats for the duration of their larval development period and so are limited in their ability to evade stress behaviourally. Carriers of the 2La inversion tend to occur in arid habitats, characterized by low rainfall and high solar radiation, therefore, these immatures are particularly prone to experience potentially stressful temperatures. The physical characteristics of water, namely high specific heat and thermal conductivity exacerbate this situation by ensuring that the organism's body temperature closely matches that of its environment. The treatments imposed in the course of this study, both TS and HH, were artificial and not intended to precisely mimic natural conditions. Rather, the intention was to provoke a response due to one aridity-linked stress: heat. As such, this study demonstrates that the 2La inversion provides its carriers with physiological tools that are better suited to larval survival in the thermally stressful environments encountered in arid habitats. Differential responses of alternative 2La arrangements to heat and other stresses linked to aridity may also be anticipated at other developmental stages (including adults) and may contribute importantly to the maintenance of the 2La polymorphism in natural populations; resolution of these questions merits further investigation.

Although no difference was found between populations in direct measures of thermo-tolerance, a significant increase in larval thermo-tolerance of 2La was revealed following heat hardening. Such a beneficial effect of heat hardening is well known in *Drosophila *[15,21] and is associated in large part with the production of heat shock proteins (HSPs) [22]. Short term exposure to a sub-lethal stress leads to an increase in the abundance of HSPs which serve among other functions as molecular chaperones for mis-folded or denatured proteins resulting from the heat stress [23]. It is very likely that differential expression of *hsp *genes is also a major source of variation in larval thermo-tolerance among alternative 2La karyotypes, a question currently under investigation.

The 2La-associated difference in the ability to induce increased thermal tolerance reveals the differential thermal sensitivity of these alternative karyotypes. Although HSP production is not threshold-dependent [24], the thermal sensitivity of HSP synthesis has been found to vary among conspecifics adapted or even acclimated to different thermal regimes [25]. In particular, organisms living in thermally variable habitats tend to be more thermally sensitive than those living in thermally stable ones [26]. Arid habitats where 2La predominates are subjected to greater diurnal temperature fluctuations than the rainforest where cloud cover buffers temperature variation. For example, puddle water temperatures on a sunny day can vary by over 10°C between morning and afternoon and tend to be higher than air temperatures by several degrees [27]. The large daily temperature fluctuations experienced in the more xeric regions of Africa probably contribute to selecting for and/or maintaining increased thermal sensitivity in carriers of the 2La inversion.

In contrast to larvae, the pupae tested were generally more resistant to the thermal stress. Pupae of *Drosophila spp*. are also more thermo-tolerant than larvae or eggs [28,29]. This difference in thermo-tolerance makes ecological sense when contrasting the mobility of larvae and pupae: in the case of fruit flies, larvae are active and may behaviourally thermoregulate, whereas pupae are immobile. Mosquito pupae are mobile, but while larvae actively move about the water column, pupae do not dive unless disturbed [30]. Also, pupation and metamorphosis are triggered by synthesis of ecdysteroids [31], hormones that are also linked to up-regulation of HSP90 synthesis [32]. Although the association of HSP90 and ecdysteroids is still unclear, the possible presence of more HSP90 during the pupal stage may be responsible at least in part for the higher thermal tolerance of pupae. In contrast to this notion, previous work with *Wyeomyia smithii *found that stress resistance decreases with developmental stage [33]. However, the nature of the stress applied to *W. smithii *was chronic rather than acute, possibly resulting in physiological changes more characteristic of an acclimation response [15].

Although pupae were generally more thermo-tolerant than larvae, HH had no effect on pupal survival following TS, regardless of karyotype. The pupal stage, during which metamorphosis occurs, is a period of intense cellular reorganization, division and differentiation. All organs are dramatically modified, most muscles are histolysed and re-synthesized [34]. An organism may respond to a heat stress by depressing routine metabolic processes to a degree dictated by its thermal sensitivity. Furthermore, increased HSP expression such as would be brought about by HH can also delay growth and cell division [35,36]. While this may be of little consequence during the larval stages, a timing glitch during the pupal stage may lead to fatal developmental abnormalities.

The gradient along which the frequency of 2La changes is characterized by variation in climatic factors, which cause variation mainly in temperature extremes rather than averages. In arid regions, where solar radiation is high, increasing morning temperatures may forecast potentially lethal afternoon conditions and warrant the induction of the stress response programme. At the opposite end of the cline, it may be disadvantageous for larvae to be thermally sensitive – the cost of mounting a stress response (e.g. energy cost, delayed growth) for small and rare temperature increases may outweigh the benefits, resulting in selection against the 2La inversion.

The differences observed among these laboratory colonies are likely modest compared to what would be observed in wild populations, as stress resistance is known to drop rapidly following laboratory adaptation [37]. Additionally, natural populations carry various combinations of chromosomal inversions that presumably interact epistatically. Before understanding the comprehensive adaptations characterizing the mosaic of natural populations, it is necessary to dissect how specific inversions affect the phenotype; this was the approach taken in the present study.

## Conclusion

Recently, genomic sequence comparison of alternative 2La karyotypes of *A. gambiae *has revealed two highly differentiated regions or "adaptation islands" representing 210 genes within the 2La inversion [17]. By comparing this gene list to results from genome-wide analyses of stress response in *D. melanogaster *[38,39], it may be possible to identify candidate genes potentially involved in stress resistance variation associated with the 2La inversion. While some of the genes identified in these adaptation islands represent promising candidates for aridity adaptation (*hsp83 *for example), it remains to be determined how they differ between alternative karyotypes and contribute to maintaining the cline observed. Combining evidence from such genotypic studies with phenotypic measurements on individuals of controlled karyotype like those presented here, will provide insights into the genes and regulatory pathways likely to be involved in inversion-associated variation in stress resistance.

The findings from this study begin to address the role of inversion polymorphisms on the phenotypic flexibility of *A. gambiae*. Over 40 inversions have been identified among populations of *A. gambiae s.s*. [3,40] and their combination in natural populations results in a multidimensional adaptive landscape of yet unknown scope [41]. Deciphering the adaptive potential of these inversions will enhance understanding of the genetic basis of physiological variation, and facilitate prediction of the effects of environmental and climate change on the distribution of this major disease vector.

## Competing interests

The authors declare that they have no competing interests.

## Authors' contributions

EG and NB conceived the study. KR performed the experiments. EG, KR and CC analysed results. EG and KR wrote the manuscript and NB edited it. All authors read and approved the final manuscript.
